# Changes of Ecosystem Service Value in a Coastal Zone of Zhejiang Province, China, during Rapid Urbanization

**DOI:** 10.3390/ijerph15071301

**Published:** 2018-06-21

**Authors:** Luodan Cao, Jialin Li, Mengyao Ye, Ruiliang Pu, Yongchao Liu, Qiandong Guo, Baixiang Feng, Xiayun Song

**Affiliations:** 1 Department of Geography & Spatial Information Techniques, Ningbo University, Ningbo 315211, China; aidushude@163.com (L.C.); yemengyao66@126.com (M.Y.); lycgeo@163.com (Y.L.); bxf3884@163.com (B.F.); 2 Donghai Institute, Ningbo University, Ningbo 315211, China; 3 School of Geosciences, University of South Florida, Tampa, FL 33620-5200, USA; rpu@usf.edu (R.P.); guo1@mail.usf.edu (Q.G.); 4 School of Accountancy, Zhejiang University of Finance and Economics, Hangzhou 310018, China; songxiayun@mail.shufe.edu.cn

**Keywords:** urbanization, coastal zone, land use, ecosystem service value (ESV), Zhejiang Province

## Abstract

Gains and losses in ecosystem service values (ESV) in coastal zones in Zhejiang Province during rapid urbanization were analyzed in terms of land-use changes. Decision-making on coastal development based on ESV estimation is significant for the sustainable utilization of coastal resource. In this study, coastal land-use changes in Zhejiang Province during rapid urbanization were discussed based on remote-sensing derived land-use maps created in the years 1990, 2000 and 2010. The ESV changes in coastal zones in Zhejiang Province from 1990 to 2010 were estimated by using the established ESV estimation model. The analysis results demonstrate the following: (1) with the continuous acceleration of urbanization, land-use types in coastal zones in Zhejiang Province changed significantly from 1990 to 2010, demonstrated by considerable growth of urban construction land and reduction of forest land and farmland; (2) in the study period, the total ESV in coastal zones in Zhejiang Province continuously decreased in value from RMB 35.278 billion to 29.964 billion, a reduction of 15.06%; (3) in terms of the spatial distribution of ESV, the ESVs in coastal zones in Zhejiang Province were generally converted from a higher ESV to a lower ESV; (4) estimates of ESV for the three years 1990, 2000 and 2010 appear to be relatively stable; and (5) land-use intensity in coastal zones in Zhejiang Province continuously increased during the 20 years. The spatial distribution of land-use intensity was consistent with that of the ESV change rate. Disordered land-use changes from forestland and farmland to urban construction land was a major cause of ESV loss.

## 1. Introduction

Research on ecosystem services can be dated back to 1864; George Marsh, an American scholar, proposed that an ecosystem can provide services to human life and activities [1]. However, his research did not attract academic attentions during the industrial age. Since the 1970s, ecosystem services have been widely emphasized in ecology [2,3]. In 1970, the Study of Critical Environmental Problems (SCEP) used for the first time “Service” in its report, Man’s Impact on the Global Environment [4]. Westman [5] proposed a concept of “nature’s services” and the evaluation for their value in 1977. In particular, natural supports for survival and development of human society were of wide concern after the concept of “sustainable development” was put forward in the United Nations Conference on Environment and Development (UNCED) in 1992, and the concept of ecosystem service was also proposed officially [6]. An ecosystem service refers to a natural utilization that is formed and maintained by an ecosystem and ecological process, on which human survival relies [7,8]. It not only offers human beings foods, medicines and other industrial and agricultural productions, but also supports and maintains life support systems on the Earth, the biogeochemical cycles and hydrologic cycles of living things, the genetic diversity of biological species, environment purification, and the atmospheric chemical balance and stability [9,10].

With rapid population growth and continuously accelerating urbanization since the industrial revolution, global ecosystems have suffered unprecedented impact and damages, resulting in a quick degradation of ecosystem services [11,12]. In 1997, Costanza et al. [13] estimated the global ecosystem service value (ESV) by constructing 17 service indices. In the same year, Daily [14] published Nature’s Services: Societal Dependence on Natural Ecosystems that not only made a systematic summarization on the concept and research history of ecosystem service and service functions of different ecosystems, but also carried out a monographic study on different ESVs. The aforementioned research events were viewed as milestones of ecosystem service studies. Thereafter, ecosystem services began to be of wide concern to ecologists, economists, sociologists and government managers across the world, and have achieved outstanding progress [15,16,17]. Existing studies of ecosystem services mainly focus on two aspects. One is to introduce and describe some concepts associated with ecosystem services and evaluation from different perspectives in order to guide current practices [18,19,20]. The other is to assess the ESV. A quantitative ESV assessment may be conducted by using different methods from different perspectives [21,22,23]. For example, the methods proposed to assess and map the ecosystem service, such as the proxy-based method, are based on expert knowledge [21,24,25]. Such methods are valuable at a large scale because collecting primary data in a large area is not feasible [26]. Another considerable method used to assess the ecosystem service is based on statistical applications of indexes to land use [13,27,28,29]. Although the statistical method has several limitations, it can directly demonstrate the economic value of the ecosystem. In addition, the calculation result in a currency form may provide a reference for the government to make a scientific decision for ecological compensation. 

Coastal zones are the transitional areas between sea and land [30]. There are complicated and diversified environmental conditions, rich natural resources, diversified ecosystems and great regional gaps in ecosystem services [13,31]. Coastal zones also play an important role in maintaining the stability of nearshore ecosystems and sustainable economic development [32,33]. Therefore, with coastal zones as a unique type of ecosystem, many scholars have studied their value and processes in those areas [31,34]. Recently, coastal zones have become regions where intensive human activities apply. Urbanization in coastal zones has been continuously accelerated with rapid coastal development and has more influence on coastal zones than natural forces do [35]. Meanwhile, with urbanization as an important symbol of land-use changes, much research interest has been paid to the influences of urbanization on ESV in coastal zones [36,37]. Such studies on ecosystem services in China began in the 1980s [38]. Recent studies on ESV in China are mainly related to evaluation factors of the ecosystem service value, proposed by Xie et al. [39], and estimating the ESVs of different regions [40,41].

Land-use change is one of the crucial driving factors of the variation of ecosystem service value [42,43]. To deal with the complex and functional land use change in a human-environment system, remote-sensing and non-remote sensing (e.g., socioeconomic) data may be integrated in spatial modeling to explore land-use dynamics [44,45,46]. The analysis of land-use change mainly focuses on analyzing dynamic changes, a driving force mechanism and corresponding environmental effects, etc. [47]. The coastal zone as a sensitive area for global change, it has an important significance for the study of profits or losses of ESV from land-use changes because of rapid urbanization [48]. ESV is an important index to evaluate impacts of land-use changes on a coastal ecological environment [49]. The ESV should be considered and assessed when a decision is made for coastal development in order to have reasonable development and utilization of coastal resources [50]. Therefore, in this study, a quantitative analysis of land-use changes in coastal zones in Zhejiang Province and the spatial-temporal change characteristics of ESV was carried out by using remote-sensing data acquired in the years 1990, 2000 and 2010 and a statistical evaluation method of ESV. The research results could be expected to provide some references for decision makers to plan reasonable coastal development and coastal ecological and environmental management as well.

## 2. Materials and Methods

### 2.1. Study Area

**Figure 1:**
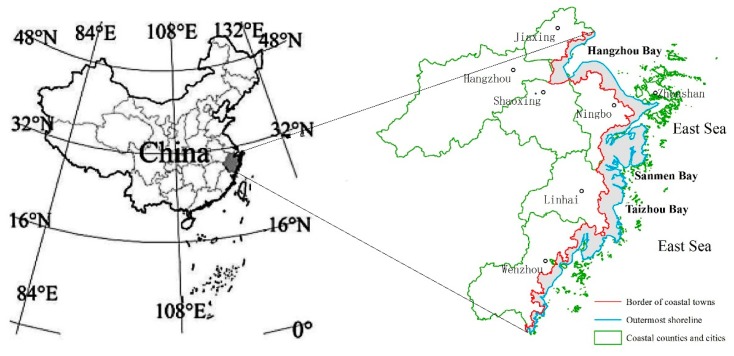
A sketch map showing the study area.

The study area (Figure 1) is located in Zhejiang Province that lies in the south of the Yangtze River Delta. Although the land area in Zhejiang Province only accounts for 1.06% of the national terrain area (101,800 km²), it possesses vast sea areas and seven coastal cities. The coastline length in Zhejiang Province is 2253.7 km. The total length of the mainland coastline and island coastline of Zhejiang Province reaches 6500 km and accounts for 20.3% of total coastline length in China [51]. The strategic concept of “One Belt and One Road” will cause profound impacts on land-use changes in the relevant regions. As a developed coastal province, Zhejiang Province plays an important role in China’s national economy [52]. The coastal zones in Zhejiang Province possess advantages of both location and transportation and are important coastal economic zones in China. With great efforts being made to become a river and ocean combined transportation service center in the new context, coastal zones in Zhejiang Province will become important nodes in the economic belt of the new Silk Road.

Coastlines in Zhejiang Province are zigzagged, with which there are various ecosystems, including reed wetland at the estuary, farmland, aquaculture land, salt pan, coastal forest land, coastal sand land and urban areas. With an accelerated process of urbanization, the land-use patterns in coastal zones in Zhejiang Province have fluctuated violently. The internal functional structure in land-use changes as well, not only destroying the ecological balance, but also threatening ecological safety and the sustainable development of society and economy.

### 2.2. Data Sources and Pre-Processing

The study area in the coastal zones in Zhejiang Province was determined by referring to the national comprehensive land-use survey of coastal zones in 1980s. The land side of coastal zones was defined as the internal boundary of coastal towns, while the sea side was defined as the external boundary after averaging the coastlines created in the years 1990, 2000 and 2010. The enclosed polygonal region formed by the boundaries landward and seaward was used as the study area (Figure 1).

Landsat TM remote-sensing images at a resolution of 30 m covering coastal zones in Zhejiang Province acquired in 1990, 2000 and 2010 were used as data sources (all image data are cloud free). Remote-sensing images were clipped by a vector layer with a spatial cover as shown in Figure 1. According to the national standard of Current Land-Use Classification, the natural ecological background and land-use status in the study area and research, the land use in the study area was categorized into seven types: forest land, farmland, construction land, water body, aquaculture land, intertidal zone and unused land. The primary land-use classification was conducted by eCognition 8.7 using a sample classification method. The three vector layers of land-use classification in the study area corresponding to the three years were created by comparative analysis and man–machine interaction interpretations.

Although there is no one-to-one match between land-use types and ecosystem types, each land use type could be linked by choosing the closest ecosystem according to existing studies [25,31,34,53,54] and actual conditions in the study area. They include the farmland ecosystem vs. farmland, forest ecosystem vs. forest land, aquatic ecosystem vs. water body, seas and aquaculture land, wetland ecosystem vs. intertidal zone, desert ecosystem vs. unused land, and artificial ecosystem vs. construction land. In this study, we merged the eight land-use types into the corresponding six ecosystems for calculating ESV.

### 2.3. Research Methods

#### 2.3.1. Land-Use Intensity Index

ESV is affected by both natural and artificial factors [55]. Coastal zones in Zhejiang Province are undergoing rapid urbanization. Large-scale urban construction has been a major factor affecting ESV changes during a relatively short time [56]. The land-use intensity not only reflects natural attributes of different land-use types themselves [57,58,59], but also shows integrative effects of human factors and natural environmental factors [60]. In this study, the land-use intensity index (I) was introduced to reflect the intensity of human activities in processes of urbanization and land-use conversion in Zhejiang Province. The formula of the land-use intensity index (I) is:

(1) $$I=\overset{n}{\underset{i-1}{\sum }}\left(L_{i}\times P_{i}\right)\times 100\%$$

where I is the land-use intensity index; a higher I represents a higher land-use intensity caused by urbanization. Li is the land-use intensity level of land-use type i, and Pi is the proportion of the land-use type i in the total land area [61]. 

Land-use types in the study area might be divided into five land-use intensity levels (L) according to the integrated land-use analysis method proposed by Zhuang et al. [61] and our practical research demands. The higher the L, the stronger the intensity of human activities. The definitions of the detailed land-use intensity levels are summarized in Table 1. 

#### 2.3.2. Ecosystem Service Value Estimation

The ESV estimation model for coastal zones in Zhejiang Province was constructed based on China’s ESV estimation model which was proposed by Xie et al. [39]. This model was also modified by referring to the ESV model proposed by Costanza [13]. Finally, the calculation formula of ESV for coastal zones in Zhejiang Province is:

(2) $$\mathrm{ESV}=\overset{n}{\underset{k-1}{\sum }}\left(A_{k}\times {\mathrm{VC}}_{k}\right)$$

where A_k_ is the area of land-use type k and VC_k_ is the ecosystem service value coefficient of land-use type k.

Originally, the ESV estimation model proposed by Xie et al. [39] that is applicable to the national scale may cause errors when directly applied to Zhejiang Province locally. For this case, the ESV coefficient of per unit area nationwide needs to be modified to establish an ESV equivalent scale for the study area [62]. The ESV coefficient is determined by referring to the relative contribution to the potential ESV and is equivalent to 1/7 of grain value/ha/year [63]. The ESV coefficient was modified accordingly. Based on Zhejiang Yearbook data, the average grain output in the study area from 1990 to 2010 was 5325.59 kg/ha and the average grain price in Zhejiang Province in 2010 was 1.967 RMB/kg. Therefore, the farmland VC in the study area was calculated as 1496.49 RMB/ha/year. Finally, value coefficients (VC) of ecosystem service for different land-use types were summarized in Table 2.

#### 2.3.3. Coefficient of Sensitivity of Ecosystem

To analyse uncertainties in the ecosystem, we examined the sensitivity of the individual land-use types to total ESV estimation [64]. The coefficient of sensitivity (CS) was used in order to highlight the importance of land-use based on their contribution to the total ESV [65]. The higher the CS value, the more important the corresponding land-use type to the total ESV. A CS of ESV can be defined as follows:

(3) $$\mathrm{CS}=\frac{{\mathrm{VC}}_{i,k}\times A_{k}}{{\mathrm{ESV}}_{i}}$$

where VC and k have the same meaning as those in Equation (2). ESV_i_ represents the initial value of ESV; the subscript i represents the original data. 

## 3. Results and Analysis

### 3.1. The Variation of Land Use

**Figure 2:**
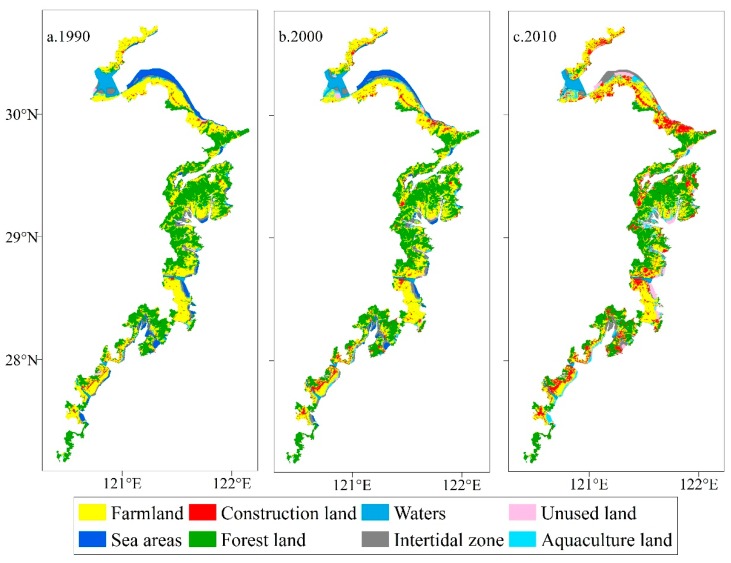
Land-use maps of the coastal zones in Zhejiang Province in the years 1990, 2000, and 2010. (**a**) the map of land-use type in the year 1990; (**b**) the map of land-use type in the year 2000; (**c**) the map of land-use type in the year 2010.

The three-year land-use maps covering the study area from 1990 to 2010 were created by interpreting Landsat TM remote-sensing images acquired in 1990, 2000 and 2010 (Figure 2). Among all land-use types, forest land and farmland had the largest distributions in the study area. Farmland was mainly concentrated in the northern plain and south-eastern coastal plain, whereas forest land was mainly located in the south-eastern coastal hilly region. Limited by geomorphology, construction land use in a scattering pattern mainly located in the northern plain and south-eastern coastal plain. In 2010, areas of forest land and farmland in the study area were 3421.47 km^2^ and 3130.43 km^2^, accounting for 34.48% and 31.55% of the total study area, respectively. The area of unused land was 322.55 km^2^ and only accounted for 3.25% of the total area. In short, in the study area the land-use intensity was high and reserved land resources are lacking (Table 3).

Significant changes of land use in the study area were observed during the study period (Table 3). Construction land, unused land and aquaculture land expanded during the 20-year period, while the remaining land-use types shrank. Construction land achieved the highest change rate (478.50%) and the corresponding area increased from 245.78 km^2^ in 1990 to 1421.81 km^2^ in 2010, which reflected a rapid progress of urbanization. Unused land showed the second highest change rate (406.91%). The area of unused land increased from 63.63 km^2^ in 1990 to 322.55 km^2^ in 2010, but it only possessed a small proportion in the total area. The area of intertidal zone increased in the first 10 years, but shrank dramatically in the second 10 years. The farmland area dropped from 3762.82 km^2^ in 1990 to 3130.43 km^2^ in 2010. The areas of forestland and water body also shrank during the same period.

### 3.2. Land-use Changes and Intensity

**Figure 3:**
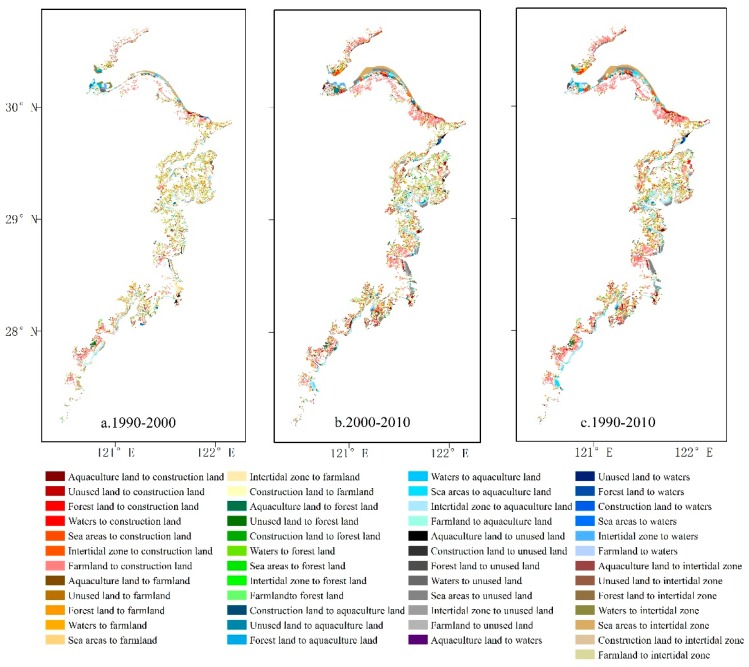
Land-use changes in the coastal zone in Zhejiang Province, 1990–2010. (**a**) land-use change from 1990 to 2000; (**b**) land-use change from 2000 to 2010; (**c**) land-use change from 1990 to 2010.

Land-use change patterns in different time periods were analyzed by using spatial analysis tools of ArcGIS (ESRI, Redlands CA, USA, available online: http://www.esri.com) in order to gain an understanding of dynamic land-use changes from 1990 to 2010 (Figure 3). The change matrix of land-use types was also constructed (Table 4).

In terms of the conversion of different land-use types, construction land expanded during the study period, which was mainly converted from farmland (861.42 km^2^). Forest land (158.46 km^2^) and the intertidal zone (55.58 km^2^) were the second major sources of construction land expansion. Although some land-use types were also converted into farmland, the increasing area (into farmland) was much smaller than its reduced area, thus resulting in a net shrinkage of farmland in general. In the same period, 198.77 km^2^ of farmland was converted into forest land, but 376.20 km^2^ forest lands were converted into farmland, finally causing a net shrinkage of forest land. The intertidal zone was mainly converted into aquaculture land (204.79 km^2^) and farmland (80.67 km^2^), showing a transformation rate of 69.86%. With rapid fishery industrial development in recent decades in Zhejiang Province, fishermen prefer to integrate coastal farmland and the new intertidal zone to increase economic benefits. Consequently, 176.24 km^2^ intertidal zones were converted into aquaculture land.

**Figure 4:**
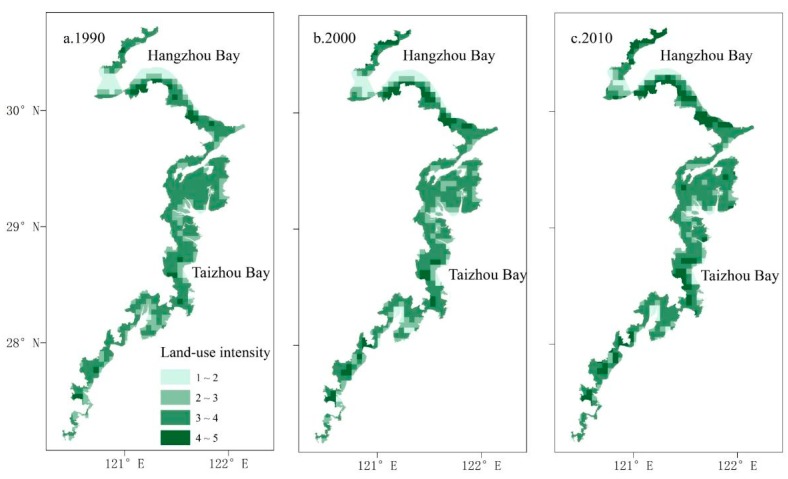
The distribution of land-use intensity for the years 1990, 2000, and 2010. (**a**) the distribution of land-use intensity for the year 1990; (**b**) the distribution of land-use intensity for the year 2000; (**c**) the distribution of land-use intensity for the year 2010.

To analyze the land-use intensity changes in coastal zones in Zhejiang Province, 5 × 5 km fishing nets were constructed by ArcGIS 10.2 to divide the study area into 636 units. The land-use intensity index of each unit was calculated (Figure 4). By comparing three maps of land-use intensity, the land-use intensity index in the study area was generally high during the 20 years and continuously increased. Land-use intensity indices of all units kept rising with the increasing rate of artificial coastal development and urbanization. The number of high-intensity units significantly increased during the 20 years, especially in coastal plains such as southern beaches in Hangzhou Bay and in Taizhou Bay.

### 3.3. Ecosystem Service Value Changes

The total ESV and ESVs of different land-use types in the study area for the three periods were calculated by using the ESV estimation model (Equation (2)). Results are listed in Table 5. The total ESV in 1990, 2000 and 2010 was RMB 35.278 billion, 34.115 billion and 29.964 billion, respectively. Forest land made the greatest contribution to the total ESV (44% ~ 48%), whereas unused land made the smallest contribution (only about 0.01%). The total ESV from 1990 to 2010 decreased by 15.06% from RMB 35.278 billion to 29.964 billion. The ESV of intertidal zone and water bodies with a relatively high VC also decreased to a certain extent.

**Figure 5:**
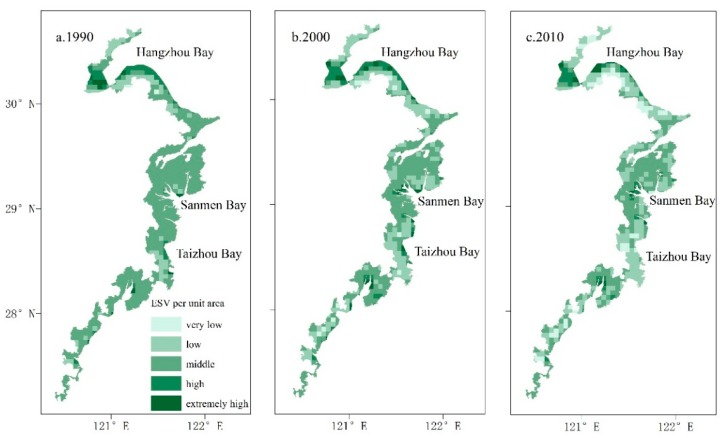
The distribution of ESV in the coastal zone in Zhejiang Province from 1990 to 2010. (**a**) the distribution of ESV in the year 1990; (**b**) the distribution of ESV in the year 2000; (**c**) the distribution of ESV in the year 2010.

The ESV of each unit was calculated by using the spatial analysis tools of ArcGIS 10.2 and was categorized into five levels as follows: very low (<RMB 10,000/ha), low (RMB 10,000/ha ~ RMB 30,000/ha), medium (RMB 30,000/ha ~ RMB 50,000/ha), high (RMB 50,000/ha ~ RMB 70,000/ha) and very high (>RMB 70,000/ha). The spatial distribution patterns of ESV for each unit are shown in Figure 5. Obviously, most units in the study area were converted from high levels to low levels. Units with a high or a very high ESV value were mainly contributed by water ecosystem at coastal water bodies (Hangzhou Bay) or sea areas. As the intertidal zone in the Hangzhou Bay was developed seawards, the ESV values of some units were changed from high value into very high value. However, the ESV of Hangzhou Bay deceased again due to rapid urbanization. Units with medium ESV were distributed widely and basically have the same distribution as forest land in coastal zones. However, the units with medium ESV became significantly narrower and were gradually converted into those with a low or very low ESV. Units with a low ESV were mainly farmland. Units with a very low ESV had the same distribution as construction land and increased dramatically because of urbanization, especially in the construction lands in Hangzhou Bay, Sanmen Bay and Jiaojiang Estuary.

**Figure 6:**
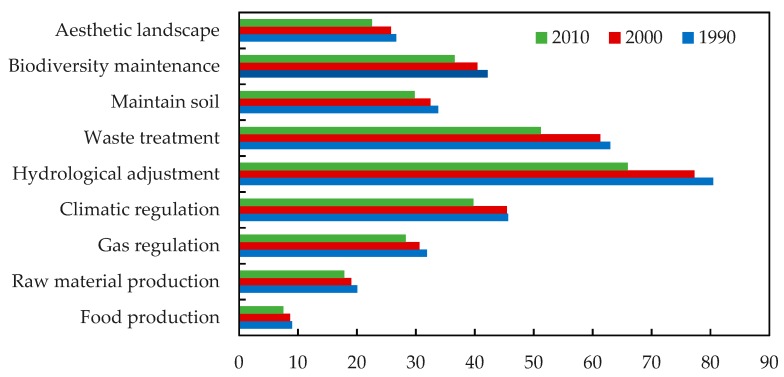
Individual ecosystem service function values (10^8^ RMB) in the study area from 1990 to 2010.

According to the ESV estimation model, individual ecosystem service function values (Figure 6) and changes (Table 6) in the study period in the three periods were calculated and presented. Hydrological adjustment, waste treatment, climatic regulation and biodiversity maintenance are principal ecosystem services in the study area.

All of these accounted for more than 10% of total ecosystem services. Since the study area is in the south-eastern coastal zone of China, which has dense water networks and adequate water bodies, the hydrological adjustment function had the highest ESV (>20% during the study period) and the food production function showed the lowest ESV (<1 × 10^9^ RMB).

It is noticeable from Table 6 that the individual ecosystem service function values decreased from 1990 to 2010. The ESVs of food production, hydrological adjustment, waste treatment and aesthetic landscape declined sharply (>15%). The ESV of waste treatment had the largest reduction (18.70%), followed by hydrological adjustment (18.02%), food production (16.13%) and the aesthetic landscape (15.47%). The ESV of raw material production had the smallest reduction (10.96%).

### 3.4. Analysis of Coefficient of Sensitivity

The Coefficient of Sensitivity of ESV was summarized in the Table 7. In the table, the CS for forest land had the largest value due to the largest VC value and the largest coverage area, which indicates that forest land is the most important in the study area when considering the total ESV. Different land-use types presented different CS. Water bodies showed a high CS value, while farmland and intertidal zone had a relative low CS value. Since the VC of unused land had only RMB 2080.12/ha/year and its area was small, the CS value of unused land was almost zero, indicating that unused land was least important to the total ESV. Additionally, although the CS values for different land-use types were various, the variation range for all CS values was ≤0.03 across the three years 1990, 2000, and 2010. In general, based on the analysis of the CS values, the estimation of ESV for the three years (1990, 2000 and 2010) appears to be relatively stable.

## 4. Discussion

### 4.1. Effects of Land-Use Changes on Ecosystem Service Value

**Figure 7:**
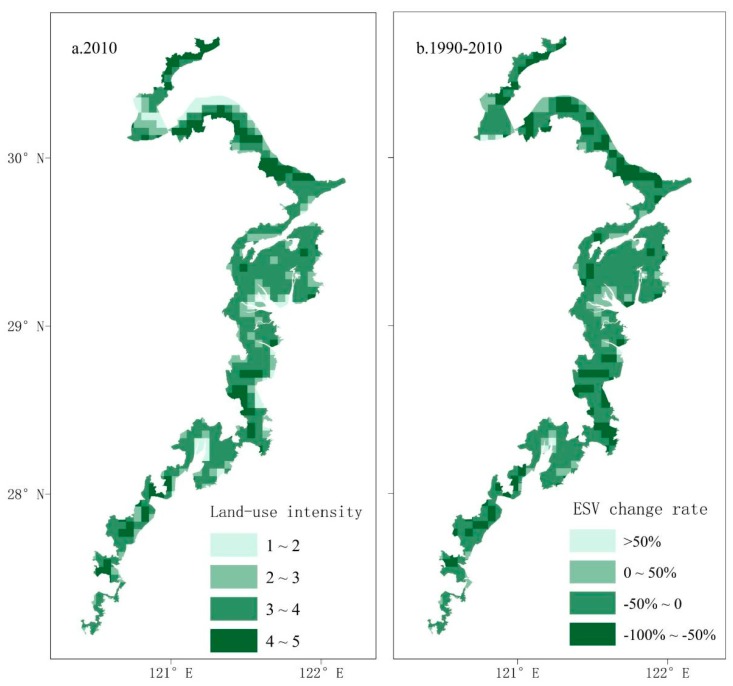
Comparison of land-use intensity with the change rate of ESV. (**a**) the map of land-use intensity in the year 2010; (**b**) the change rate of ESV from 1990 to 2010.

The spatial distribution of land-use intensity in 2010 was consistent with the spatial distribution of ESV change rate from 1990 to 2010 (Figure 7). The ESVs of regions with a high land-use intensity index significantly reduced during the 20 years, indicating that the land-use changes influenced ESV significantly because of urbanization. Land-use changes are closely related to ecosystem services [66]. Firstly, the land is the carrier of various terrestrial ecosystems. Land-use changes will cause changes of land type, area and spatial position. Different land-use types offer different principal ecosystem services. Thus, land-use changes result in differences in type, area, value and spatial pattern of different ecosystems [67]. Secondly, land-use changes are accompanied by changes of the ecological process and lead to the changes of ecosystem services and ESV through the interaction of multiple factors [68]. Furthermore, different land-use intensities have different influences on ecosystem services. 

### 4.2. Causes of Ecosystem Service Value Changes in the Study Area

Zhejiang possesses tremendous agricultural production elements and stable natural conditions that marginally affect the land-use types [69]. Land-use changes directly result from rapid economic development, urbanization, industrialization and population growth [70,71]. Rapid urbanization increased the area of construction land, but shrank that of forest land and farmland significantly. Industrial structure adjustment, especially the annual growth of aquaculture land area, took a toll on water body and intertidal zone areas. Land-use changes in the study area influenced ESVs of different land-use types directly. From 1990 to 2000, the shrinkage of forest land and water body was the main cause of ESV reduction. Expansion of intertidal area led to ESV reduction. From 2000 to 2010, the shrinkage of water body, intertidal zone, farmland and forest land reduced ESV considerably.

The individual ecosystem service function value decreased with the increase of land-use intensity. During rapid urbanization, the areas of farmland, nearshore sea area and intertidal zone decreased because of construction land expansion and sea reclamation. Land-use changes may cause different impacts on different ecosystems. For example, the adjustment service and supply service of an ecosystem are closely related to the area of land-use types. Ecological services such as supply service and adjustment service were weakened with the area shrinkage [11]. The higher ESV regions are distributed in the coastal water ecosystems, such as Hangzhou Bay, Taizhou Bay and Sanmen Bay. However, the areas of the higher ESV regions were gradually decreased during the development of urbanization from 1990 to 2010. In recent years, given the importance of the ecosystem in coastal zones, local governments in China have set out a series of policies such as “General Plan for Intertidal Zone in Zhejiang Province, 2005~2020”. The Plan stipulated that an intertidal zone of the bay must be protected in Zhejiang coastal zone during urbanization, especially for wetlands. In terms of the CS values (Table 7), which can reflect the contribution of a land-use type to the total ESV, the land-use type: forest land, waters, and intertidal zone are important to the total ESV in our study. Therefore, strict ecological planning should be carried out to limit the intensity of human activity, and the development of land-use types of forest land, waters, and intertidal zones should be encouraged in future in order to protect the coastal ecological environment and increase total ESV. Therefore, understanding the land-use change process and impact factors during urbanization can not only provide scientific references for the optimization of land-use patterns, but also ensure ESV value effectively. It is beneficial to ecological environmental restoration and promotes scientific management for the coastal ecological environment and sustainable social and economic development. Governments shall make a detailed plan to guide urbanization in order to protect the coastal ecological environment and increase the ESV. Land-use changes caused by rapid urbanization change the ESV directly and influence the ESV changes indirectly through the interaction of factors that cause land-use changes. Hence, future studies need to explore the relationship and interactive mechanism between land-use types and the ESV.

### 4.3. Limitations of the Ecosystem Service Value Estimation Model

The results obtained in this study in estimating total ESV and the ESV’s composition might suffer from larger errors compared to actual values. Firstly, the ESV estimation model does not involve genetic resource supply, disease control, natural disaster control and spirit and religions due to the limitation of evaluation methods and data availability. Thus, the total ESV might be underestimated using the model. Moreover, the ecosystem category in this study has some shortages. Because not all land-use types have corresponding ecosystems, the ESV of each land-use type is estimated based on the most similar ecosystem. Secondly, most parameters in constructing the model were empirically estimated based on the data collected from relevant studies, which could introduce some errors. Finally, the study of coastal zones in Zhejiang Province is at a local scale. The areas of ecological land-use types such as farmland, forest land and water bodies are relatively small, thus resulting in a lower total ESV. For instance, the global ESV calculated by Costanza et al. [13] was 1.8 times of that of global GDP, whereas the ESV of coastal zones in Zhejiang Province in 2010 (RMB 29.964 billion) was only equivalent to 1.88% of GDP (RMB 1590.572 billion) of that year.

## 5. Conclusions

Land-use types and total ESV in coastal zones in Zhejiang Province from 1990 to 2010 were analyzed and estimated by remote-sensing and geographic information system (GIS) techniques according to the terrestrial ESV factors in China. From the study, several conclusions were derived as follows:

(1) Farmland was mainly concentrated in the northern plain and south-eastern coastal plain in the study area, while forest land mainly lay in south-eastern coastal hilly regions. Land-use types changed significantly from 1990 to 2010 due to intensive human activities, proved by dramatic construction land expansion and large shrinkage of forest land and farmland. 

(2) In the study period, the expanded construction land resulted mainly from farmland (861.42 km^2^). The second and third resources were forest land and intertidal land. The total area of farmland and forest land decreased during the 20 years. The intertidal zone was mainly converted into farmland (69.86%). The land-use intensity index in the study area kept increasing continuously with the acceleration of urbanization, especially in coastal plains like the south beach of Hangzhou Bay and Taizhou Bay.

(3) The total ESV in coastal zones in Zhejiang Province decreased from RMB 35.278 billion to 29.964 billion, showing a reduction of 15.06%. Hydrological adjustment, waste treatment, climate regulation and biodiversity maintenance have been prominently affected in the study area. In addition, individual ecosystem service function values kept decreasing continuously during the past decades, indicating an obvious degradation of the ecological environment.

(4) Units with a higher ESV in the study area changed to those with a lower ESV during the time period, especially in coastal construction lands in Hangzhou Bay, Sanmen Bay and Jiaojiang Estuary. The land-use change caused by disordered expansion of construction land is the major cause of the ESV loss.

(5) The land-use intensity index in coastal zones in Zhejiang Province kept increasing in the study period. The spatial distribution of the land-use intensity index value was consistent with the spatial distribution of the ESV change rate. Urbanization in the study area intruded ecological land resources, including farmland and forest land. This directly causes ecosystem degradation and considerable ESV reduction.

## Figures and Tables

**Table 1:** Levels of land-use intensity.

Intensity Level	Land-Use Type	Value
Unused level	Unused land and intertidal zone	1
Light utilization level	Waters	2
Low utilization level	Forest land	3
Strong utilization level	Farmland	4
High-strength utilization level	Construction land	5

**Table 2:** Value coefficients (RMB/ha/year) of ecosystems service.

Ecosystem Service and Function		Forest Land	Farmland	Intertidal Zone	Waters	Unused Land	Construction Land
Supply service	Food production	493.84	1496.49	538.74	793.14	29.93	0.00
	Raw material production	4459.54	583.63	359.16	523.77	59.86	0.00
Regulating service	Gas regulation	6464.84	1077.47	3606.54	763.21	89.79	0.00
	Climatic regulation	6090.71	1451.60	20,277.44	3082.77	194.54	0.00
	Hydrological adjustment	6120.64	1152.30	20,112.83	28,089.12	104.75	0.00
	Waste treatment	2573.96	2080.12	21,549.46	22,222.88	389.09	0.00
Support services	Soil conservation	6015.89	2199.84	2978.02	613.56	254.40	0.00
	Biodiversity maintenance	6749.17	1526.42	5522.05	5132.96	598.60	0.00
Cultural services	Aesthetic landscape	3112.70	254.40	7018.54	6644.42	359.16	0.00
Total		42,081.30	11,822.27	81,962.76	67,865.82	2080.12	0.00

**Table 3:** The changes of land use in the coastal zones in Zhejiang Province from 1990 to 2010.

Year	1990	2000	2010	1990–2000		2000–2010		1990–2010	
Land-Use Type	Area/km^2^			Variation of Land Use					
				Area/km^2^	rate/%	Area/km^2^	rate/%	Area/km^2^	rate/%
Farmland	3762.82	3664.51	3130.43	−98.31	−2.61	−534.08	−14.57	−632.39	−16.81
Sea areas	767.61	529.72	0.00	−237.89	−30.99	−529.72	−100.00	−767.61	−100.00
Construction land	245.78	522.34	1421.81	276.56	112.52	899.49	172.20	1176.05	478.50
Forest land	3788.64	3576.25	3421.47	−212.39	−5.61	−154.78	−4.33	−367.17	−9.69
Waters	518.40	457.17	422.22	−61.23	−11.81	−34.95	−7.64	−96.18	−18.55
Intertidal zone	625.50	703.39	540.65	77.89	12.45	−162.74	−23.14	−84.85	−13.57
Unused land	63.63	138.68	322.55	75.05	117.95	183.87	132.59	258.92	406.91
Aquaculture land	150.04	330.36	663.29	180.32	120.18	322.93	97.75	503.25	335.41

**Table 4:** The change matrix of land use in the coastal zone in Zhejiang Province from 1990 to 2010.

Area/km^2^		Year 2010								
		Farmland	Construction Land	Forest Land	Water Body	Intertidal Zone	Unused Land	Aquaculture Land	Total	Change Rate/%
**Year 1990**	**Farmland**	2453.22	861.42	198.77	38.78	18.23	13.59	176.24	3760.24	34.76%
	**Sea areas**	60.48	50.20	4.05	30.37	278.76	199.49	140.75	764.12	100.00%
	**Construction land**	35.90	193.21	9.82	4.40	0.45	0.80	0.98	245.56	21.32%
	**Forest land**	376.20	158.46	3173.52	19.76	11.87	19.78	23.58	3783.18	16.11%
	**Water body**	81.79	40.87	4.13	303.99	37.05	2.13	47.99	517.95	41.31%
	**Intertidal zone**	80.67	55.58	12.50	20.97	187.96	61.25	204.79	623.74	69.86%
	**Unused land**	9.06	20.74	13.17	1.11	1.13	7.66	11.41	64.28	88.09%
	**Aquaculture land**	30.17	39.26	0.90	2.76	2.73	17.34	56.48	149.64	62.26%
	**Total**	3127.49	1419.73	3416.87	422.15	538.18	322.05	662.21	9908.70	

**Table 5:** Changes of ecosystem service value (ESV) in the coastal zone in Zhejiang Province from 1990 to 2010.

Land-Use Type	ESV/10^8^ RMB/year			ESV Change/10^8^ RMB/year					
	1990	2000	2010	1990–2000	Change Rate/%	2000–2010	Change Rate/%	1990–2010	Change Rate/%
Forest land	159.43	150.49	143.98	−8.94	−5.61	−6.51	−4.33	−15.45	−9.69
Farmland	44.49	43.32	37.01	−1.17	−2.63	−6.31	−14.57	−7.48	−16.81
Intertidal zone	51.27	57.65	44.31	6.38	12.44	−13.34	−23.14	−6.96	−13.58
Waters	97.46	89.4	73.67	−8.06	−8.27	−15.73	−17.60	−23.79	−24.41
Unused land	0.13	0.29	0.67	0.16	123.08	0.38	131.03	0.54	415.38
Construction land	0.00	0.00	0.00	0.00	0.00	0.00	0.00	0.00	0.00
Total	352.78	341.15	299.64	−11.63	−3.30	−41.51	−12.17	−53.14	−15.06

**Table 6:** Changes of structures of ESVs in the coastal zone in Zhejiang Province from 1990 to 2010.

Ecosystem Service Function	1990–2000		2000–2010		1990–2010	
	Functional Value Change (10^8^ RMB)	Change Rate/%	Functional Value Change (10^8^ RMB)	Change Rate/%	Functional Value Change (10^8^ RMB)	Change Rate/%
Food production	−0.31	−3.45%	−1.14	−13.13%	−1.45	−16.13%
Raw material production	−1.03	−5.13%	−1.17	−6.14%	−2.20	−10.96%
Gas regulation	−1.28	−4.01%	−2.32	−7.58%	−3.60	−11.29%
Climatic regulation	−0.21	−0.46%	−5.69	−12.52%	−5.90	−12.92%
Hydrological adjustment	−3.18	−3.95%	−11.32	−14.65%	−14.50	−18.02%
Waste treatment	−1.69	−2.68%	−10.09	−16.46%	−11.78	−18.70%
Maintain soil	−1.32	−3.90%	−2.68	−8.24%	−4.00	−11.82%
Biodiversity maintenance	−1.72	−4.08%	−3.84	−9.49%	−5.56	−13.18%
Aesthetic landscape	−0.90	−3.37%	−3.23	−12.52%	−4.13	−15.47%

**Table 7:** The sensitivity index of the ESV coefficient.

Land-Use Type	CS		
	1990	2000	2010
Forest land	0.45	0.44	0.48
Construction land	0.00	0.00	0.00
Farmland	0.13	0.13	0.12
Intertidal zone	0.15	0.17	0.15
Waters	0.28	0.26	0.25
Unused land	0.00	0.00	0.00
